# Use of powered air-purifying respirator (PAPR) by healthcare workers for preventing highly infectious viral diseases—a systematic review of evidence

**DOI:** 10.1186/s13643-020-01431-5

**Published:** 2020-08-08

**Authors:** Ana Licina, Andrew Silvers, Rhonda L. Stuart

**Affiliations:** 1 Austin Health, Heidelberg, Australia; 2 Monash Medical Centre, Clayton, Australia; 3 Faculty of Medicine, Nursing and Health Sciences, Monash University, Melbourne, Victoria Australia; 4 Infection Prevention & Epidemiology, Monash Health, Clayton, Victoria Australia

**Keywords:** SARS-CoV-2, SARS-CoV-1, Powered air-purifying respirator, Respiratory protection, Healthcare worker

## Abstract

**Background:**

Healthcare workers (HCWs) are at particular risk during pandemics and epidemics of highly virulent diseases with significant morbidity and case fatality rate. These diseases include severe acute respiratory syndrome coronaviruses, SARS-CoV-1 and SARS-CoV-2, Middle Eastern Respiratory Syndrome (MERS), and Ebola. With the current (SARS-CoV-2) global pandemic, it is critical to delineate appropriate contextual respiratory protection for HCWs. The aim of this systematic review was to evaluate the effect of powered air-purifying respirators (PAPRs) as part of respiratory protection versus another device (egN95/FFP2) on HCW infection rates and contamination.

**Methods:**

Our primary outcomes included HCW infection rates with SARS-CoV-2, SARS-CoV-1, Ebola, or MERS when utilizing PAPR. We included randomized controlled trials, non-randomized controlled trials, and observational studies. We searched the following databases: MEDLINE, EMBASE, and Cochrane Library (Cochrane Database of Systematic Reviews and CENTRAL). Two reviewers independently screened all citations, full-text articles, and abstracted data. Due to clinical and methodological heterogeneity, we did not conduct a meta-analysis. Where applicable, we constructed evidence profile (EP) tables for each individual outcome. Confidence in cumulative evidence for each outcome was classified according to the GRADE system.

**Results:**

We identified 689 studies during literature searches. We included 10 full-text studies. A narrative synthesis was provided. Two on-field studies reported no difference in the rates of healthcare workers performing airway procedures during the care of critical patients with SARS-CoV-2. A single simulation trial reported a lower level of cross-contamination of participants using PAPR compared to alternative respiratory protection. There is moderate quality evidence that PAPR use is associated with greater heat tolerance but lower scores for mobility and communication ability. We identified a trend towards greater self-reported wearer comfort with PAPR technology in low-quality observational simulation studies.

**Conclusion:**

Field observational studies do not indicate a difference in healthcare worker infection utilizing PAPR devices versus other compliant respiratory equipment. Greater heat tolerance accompanied by lower scores of mobility and audibility in PAPR was identified. Further pragmatic studies are needed in order to delineate actual effectiveness and provider satisfaction with PAPR technology.

**Systematic review registration:**

The protocol for this review was prospectively registered with the International Register of Systematic Reviews identification number CRD42020184724.

## Background

High infectivity combined with high case fatality rate during the COVID-19 pandemic has placed an emphasis on healthcare worker (HCW) protection both from a personal as well as a societal perspective. Several other outbreaks of virulent highly infectious diseases have occurred in recent decades including the Ebola crisis in 2014–2016, Middle East respiratory syndrome coronavirus (MERS-CoV), and severe acute respiratory syndrome (SARS, due to SARS-CoV-1) epidemic [1, 2]. Teasing out the true infection risk in the HCW group is difficult. This is due to the high rates of community infection, HCW travel and under-reporting of non-HCW populations, and the lack of phylogenetic viral analysis. Personal protective equipment (PPE) and infection control guidelines from the WHO are based on the assumption that the primary mechanism of transmission of SARS-CoV-2 is direct and indirect droplet spread as well as fomite transmission [3]. The WHO advises that airborne transmission can occur, but only when aerosol-generating procedures (AGPs) are performed in patients infected with SARS-CoV-2 [4]. Aerosol-generating procedures result in the generation of small aerosolized particles through disruption of surface tension of the alveolar lining [5]. Aerosolized particle clouds can travel up to 8 m [6]. A detailed list of AGPs is provided in Table 1 [7]. The degree of airborne spread in the coronavirus group is contentious [8, 9]. Recently, the stability of SARS-Cov-2 and SARS-Cov-1 was evaluated under different experimental conditions [10]. SARS-CoV-2 and SARS-CoV-1 remained viable in aerosols throughout the 3-h duration of the experiment with a reduction in infectious titer [10]. However, the clinical relevance of this experimental model has been questioned [11]. Establishing with certainty whether SARS-CoV-2 is infectious through airborne transmission may be methodologically challenging.

**Table 1:** List of aerosol-generating procedures (AGPs)

*Respiratory aerosols*	*Blood or tissue fluid aerosols*
Open suctioning of airways	Surgical procedures in which high-speed tissue drills are used in the airway (e.g., ear nose and throat surgery, head and neck surgery)
Sputum induction	Extensive dental procedures
Bronchoscopy and bronchoalveolar lavage	
Endotracheal intubation and extubation	
Face-mask ventilation	
Non-invasive ventilation (e.g., BiPAP, CPAP)	
Ventilation when the airway is not sealed	
Tracheostomy	
Cardiopulmonary resuscitation	
Nasogastric tube insertion	
Dental drilling procedures	

Abbreviations: *BiPAP* bilevel positive ventilation pressure, *CPAP* continuous positive airway pressure

In this review, we consider the implication for HCWs of Ebola in addition to the coronaviruses. Ebola virus can be transmitted by direct contact with blood, bodily fluids, or skin of Ebola patients or individuals who have died of the disease. Development of Ebola disease results in a high case fatality rate, as high as 50%. Recommendations for respiratory protective equipment are therefore similar [12].

### Description of the devices

Two major international testing and classification bodies of respiratory protection include the National Institute for Occupational Safety and Health (NIOSH) and European Norms (EN). Air-purifying particulate respirators function by removing aerosols (droplets and solid particles) from the air through the use of filters, cartridges, or canisters. Air-purifying respirators fall into one of four different classifications (Table 2): (1) filtering facepiece respirator (FFR), (2) elastomeric half facepiece respirator, (3) elastomeric full facepiece respirator, and (4) powered air-purifying respirator (PAPR). The two major testing institutions (NIOSH and EN) employ different test protocols for the evaluation of air-purifying particulate respirators as well as having different nomenclatures (Table 2). In the USA, respiratory filtration levels are determined according to the Occupational Health and Safety Administration (OSHA) standard 29 CFR 1910.134 “Respiratory Protection” [13]. Meanwhile, the EN requires 94 and 99% efficiencies for FFRs, class P2 (FFP2), and class P3 (FFP3), respectively [14]. In Europe, respirators are tested against the relevant European Standard and are approved to the PPE Directive 89/686/EEC or the replacement PPE Regulation (EU)2016/425 [15].

**Table 2:** Filtering facepiece, air-purifying respirator(APR) and powered air-purifying respirator (PAPR) classification according to NIOSH/EN (National Institute for Occupational Safety and Health (NIOSH) and European Norms (EN) with stated assigned protection factor (APF)

Respirator type	NIOSH nomenclature	EN nomenclature	Minimum filtration capacity for particles > 0.3 microns	OSHA APF	EN Standard APF
Face filtering respirator		FFP1	80%		4 fold
		FFP2	94%		10 fold
	N95		95%	10 fold	
	N99	FFP3	99%	10 fold	20 fold
	P100		99.97%	10 fold	20 fold
Air-purifying respirator (APR)	APR half facepiece	APR half facepiece	As per selected filter	10	10
Air-purifying respirator (APR)	APR full facepiece	APR full facepiece	As per selected filter;	10–50	10–50
Powered air-purifying respirator (PAPR)	PAPR half facepiece	PAPR half facepiece	99.97%	50	50
	PAPR full facepiece	PAPR full facepiece	99.97%	1000	1000
	PAPR helmet/hood	PAPR helmet/hood	99.97%	25–1000	25–1000
	Loose-fitting facepiece	Loose-fitting facepiece	99.97%	25	25

Explanation: Please note: “Minimum filtration capacity tends to be a unified measure for any and all particles whether biological or particulate”

The assigned protection factor (APF) of a respirator denotes the level of protection that the respirator is expected to provide to users who are properly fitted and trained. The APF is the ratio of pollutants outside the device (environment) to that inside the device (inhaled component). For example, an APF of 10 “means that a user could expect to inhale no more than one tenth of the airborne contaminant present.” Airborne level protection includes helmets, covers, and hoods; FFP3 or FFP2/N95 masks, goggles, or face shields (if no helmets).

PAPRs can be described as respirators that protect the user by filtering out contaminants in the air and use a battery-operated blower to provide the user with clean air through a tight-fitting respirator, a loose-fitting hood, or a helmet. There is a wide heterogeneity of the available PAPR devices. Traditional PAPRs used in healthcare settings have a full-facepiece and loose-fitting hoods, attached to waist-mounted belt batteries. PAPRs use the high-efficiency particulate air (HEPA) filters and provide a higher level of protection than disposable respirators. High-efficiency particulate air (HEPA) filters have a similar filtration as P100 (i.e., they filter at least 99.97% of particles 0.3 μm in diameter and are oil-proof [9]. PAPRs are considered more protective in terms of the level of respiratory protection due to the higher efficiency of their filtration pieces as well as the maintenance of outward positive pressure. PAPRs are specified for high-hazard procedures because they can offer assigned APFs ranging from 25 to 1,000, which reduce the risk more than the protection factors provided by N95 respirators. The improved protection is largely provided by the positive pressure in the head covering or facepiece (Table 2). The hoods of PAPRs can provide splash protection and some degree of eye protection [14, 16]. If HCWs are provided with sufficient comfortable and well-fitting respiratory protection, it is likely that compliance with preventive programs will be increased [17].

### How the intervention might work

In the first instance, relevant individual institutions need to safeguard an HCW respiratory compliance program. An appropriate choice of the level of respiratory protection needs to be made within this program.

There is a significant heterogeneity of international recommendations with regard to appropriate respiratory protection for HCWs when performing AGPs in suspected or confirmed COVID-19 patients is notable. The European Centre for Disease Prevention and Control (ECDC) prevention and Centre for Disease Control (CDC) USA recommend the use of an at least N95/FFP2 and a higher level of protection [7, 18] the Public Health England recommends FFP3 level respiratory protection in addition to standard PPE [19], the Communicable Disease Network Australia (CDNA) recommends FFP2/N95 mask, and with regard to the use of PAPR, CDNA recommends that if a healthcare worker is required to remain in the room for longer periods of time (greater than 1 h), the use of PAPR may be considered for additional comfort and visibility [20] (Table 3).

**Table 3:** International recommendations of respiratory component of PPE for protection of HCWs performing AGPs in suspected or conformed COVID-19 patients

International governing body/institution	Face filtering piece (FFP) (in addition to other PPE)	Powered air-purifying respirator (PAPR) (in addition to other PPE)
European Centre for Disease (ECDC)	FFP2/FFP3	Use of PAPR not considered
Centers for Disease Control and Prevention (CDC)	At least N95	Use of PAPR not considered
Public Health of England	FFP3	Use of PAPR not considered
The Communicable Disease Network Australia (CDNA)	FFP/N95	Consider the use of PAPR if remaining in the room with patient with suspected/confirmed COVID-19 positive patient longer than 1 h

*Abbreviations: FFP* face filtering piece, *PPE* personal protective equipment, *HCW* healthcare workers, *AGP* aerosol-generated procedures

### Why it is important to do this review

Evidence-guided practice for the respiratory component of personal protective equipment is limited. With the high rate of HCW infection during the SARS-COV-1 epidemic in Toronto, the PAPR use became embedded in respiratory protocols [21, 22]. Limited information exists for use of one type of facial protection (e.g., FFP3) over another (e.g., FFP2/N95). High filtration pieces appear to have a protective advantage in laboratory settings [23]. However, this does not translate to firm findings of greater healthcare worker protection in field studies [24]. Increased layers and technical challenges of personal protective equipment can lead to the increased complexity of patient care [25]. During outbreaks such as the current global pandemic, early recommendations are often based on precautionary principles. It is uncertain what level of respiratory protection is required routinely for aerosol-generating procedures (AGPs) in highly infectious viral diseases as evidenced by heterogenous international recommendations. In 2005, Yassi et al. identified the recommended level of respiratory protection as a critical gap in societal understanding of viral pandemic management [26]. There are known logistical advantages and disadvantages to PAPR technology (see Table 4).

**Table 4:** Logistical advantages and disadvantages of PAPR, adapted from Wong et al. [27]

*Advantages of PAPR*	*Disadvantages of PAPR*
PAPRs use only HE filters, which have a greater filtration efficiency against the smallest pathogen particles compared to face-filtering respirators (FFRs)	Challenges in verbal communication
PAPR systems have assigned protection factors (APF) of at least 25	May limit the visual field
Provides eye protection (hooded models only)	Inability to auscultate chest
PAPRs with loose-fitting headgear can be worn with a limited amount of facial hair	Proper maintenance of PAPR requires disinfection, cleaning, safe storage, and battery maintenance
	Inability to re-use disposable filters between patients (need a large supply of filters)
	Risk of battery failure and inadvertent exposure
	Requires decontamination after use
	More expensive than individual N95 respirator (although achieve more wears per piece of equipment with PAPR)
	Requirement for the education of a significant proportion of HCW workforce

We aim to summarize and critically appraise current evidence of the effectiveness of PAPR for preventing nosocomial infection in health care staff exposed to respiratory/body fluids contaminated with highly infectious viral diseases such as SARS-CoV-2, SARS-CoV-1, MERS, and Ebola. In particular, we will try and address current questions identified from the COVID-19 epidemic that include to what effect PAPR as part of respiratory protection versus another (e.g., N95/P2) has on HCW infection rates and contamination.

## Methods

Our findings have been reported according to the standards for the Preferred Reporting Items for Systematic Reviews and Meta-Analysis [28]. The protocol for this review was prospectively registered with the International Register of Systematic Reviews identification number CRD42020184724.

### Eligibility criteria

#### Types of studies

We included randomized controlled trials which compared different types of PAPR, whether reusable or disposable, for the prevention of HCW infection. We included observational studies, defined as studies that follow HCWs over time and that compare the effect of PAPR. We included simulation studies of PAPR technology or alternative respiratory equipment for donning and doffing procedures. In order to maximize study capture, we have chosen a broad range of applicable methodological approaches.

Our full eligibility criteria are listed in Table 5.

**Table 5:** Review eligibility criteria

*Study characteristic*	*Inclusion criteria*	*Exclusion criteria*
Types of participants	Healthcare workers volunteers	
Intervention treatment	Powered air-purifying respirator (PAPR) studied separately or within a personal protective equipment (PPE).	Hybrid PAPR (hybrid PAPR is designed as both a self-contained breathing apparatus, PAPR and a standard mask—their design features may not reflect a true PAPR device intended for healthcare use)
Comparator	Any other respiratory protective equipment, FFP3/FFP2/N95, or surgical masks.	
Outcomes	-Healthcare worker infection rates utilizing PAPR technology within a PPE program as infection with SARS-Cov-2, SARS-Cov-1, EBOLA, or MERS; -Contamination of skin or clothing measured with any type of test material to visualize contamination; -Compliance with guidance on the use of PAPR measured with, e.g., observation checklist; -Level of wearer comfort whilst using the PAPR; -Objective and/or subjective measures of work of breathing during the use of PAPR; -Costs of resource use of PAPR equipment; -Impact of structured training programs on PAPR use;	
Study design	Randomized controlled trials Non-randomized studies Observational studies (cohort studies, case-control studies, cross-sectional studies, case series)	Case reports Surveys
Study setting	Inpatient care/critical care/intensive care;	
Timing	Perioperative process-preadmission, preoperative, intraoperative, and postoperative setting	Studies incorporating long-term (greater than 3 months) postoperative rehabilitation

#### Types of participants

For simulation studies, we included any type of participants (volunteers or HCW) using PAPR or alternative respiratory equipment as part of a protective PPE program. For field studies, we planned to include any HCW exposed to body fluids from patients contaminated with Ebola, MERS, SARS-Cov-1, or SARS-Cov-2.

#### Types of interventions

We included studies that evaluated the effectiveness of any type of purified airflow respirator (PAPR), whether disposable or recyclable against suitable face respirators such as N95/FFP2 or any other respiratory protection used. We excluded hybrid PAPR devices due to the potential for confounding.

#### Types of outcome measures

##### Primary outcomes

We planned to include all eligible studies that have measured:

1. Healthcare worker infection rates utilizing PAPR technology within a PPE program for infection with SARS-Cov-2, SARS-Cov-1, EBOLA, and MERS;
2. Contamination of the skin or clothing measured with any type of test material to visualize contamination;
3. Compliance with guidance on the use of PAPR measured with, e.g., observation checklist;

##### Secondary outcomes

We planned to include all eligible measures that have measured the following:

1. Level of wearer comfort, visibility, and audibility whilst using the PAPR over alternative respiratory protection;
2. Objective and/or subjective measures of work of breathing during the use of PAPR versus alternative respiratory protective equipment;
3. Costs of resource use including maintenance and cleaning of PAPR equipment;
4. Impact of structured training programs on PAPR use over alternative training or no teaching;

### Information sources and literature searches

We searched the following electronic databases: MEDLINE via Ovid SP, EMBASE via Ovid SP, and Cochrane Library (Cochrane Database of Systematic Reviews and CENTRAL). In addition, we sought information from gray literature through the following specific search engines: Google Scholar, OpenGrey, and GreyNet [29–31]. We developed a search strategy for MEDLINE via Ovid (Additional file 1) and adapted it for other databases. We searched all databases from their inception to the present time. We conducted the original search for studies in May 2020. Due to the dynamic nature of the current pandemic, we repeated our searches in June 2020. We limited our search to English language studies. We did this in order to facilitate the efficiency of the search, bearing in mind that language limitation is unlikely to result in publication bias [32].

### Study selection

**Fig. 1:**
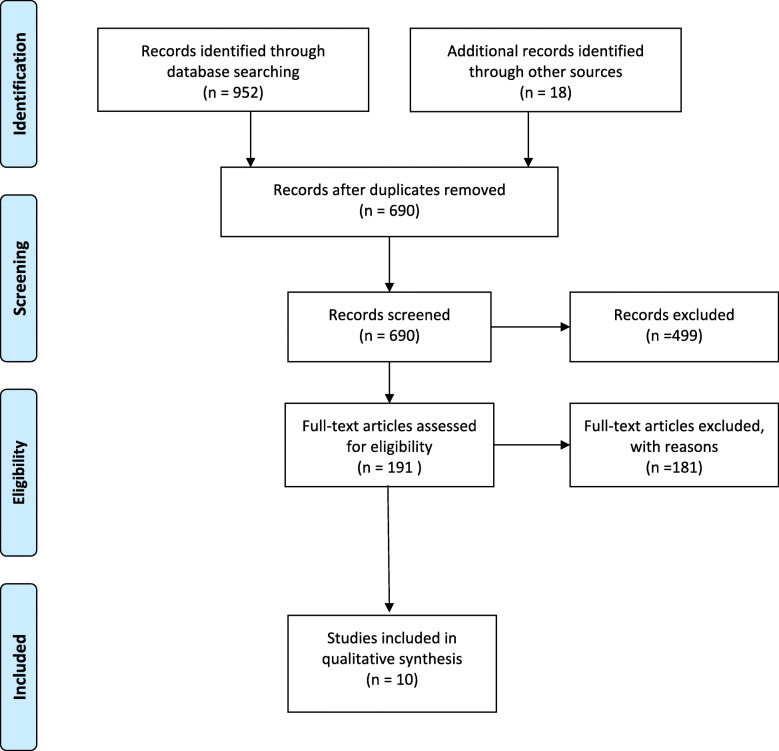
PRISMA flow diagram

Titles and abstracts of articles returned from initial searches were screened by two reviewers *(AL)* and *(AS)* based on the eligibility criteria outlined above. Full texts of potential eligible studies were examined for suitability. References of all considered articles were hand-searched to identify any other potentially eligible studies. Any disagreements were resolved by discussion. The results of the data search were presented in a PRISMA flow diagram indicating the number of studies retrieved, screened, and excluded as per exclusion criteria (see Fig. 1).

### Data extraction, management, analysis, and presentation

Data were extracted from each study including publication details, study characteristics, participant characteristics, type of procedure, intervention and comparator characteristics, and outcomes. For randomized controlled trials, one author *(AL)* extracted the information on the methodological quality of studies including random sequence generation, allocation concealment, blinding of participants, and personnel, blinding of outcome assessment, incomplete outcome data, selective outcome reporting, and other bias [33]. For non-randomized studies, data were collected on all applicable elements other than random sequence generation and allocation concealment.

### Risk of bias in individual studies

The risk of bias in randomized controlled studies was assessed using the Cochrane Risk of Bias tool [34]. We used the ROBINS-I (Risk of Bias in Non-randomized Studies of Interventions) tool to assess the risk of bias in non-randomized studies [35]. We rated each potential source of bias as high, low, or unclear. We considered blinding separately for different key outcomes where necessary. We used the risk of bias assessment in individual studies to inform our assessment of study limitations across the body of evidence.

### Data synthesis

We planned to systematically describe the data from each study. We planned to generate the evidence profile table across each predetermined primary and secondary outcome. We planned to pool data from studies judged to be clinically homogeneous using Review Manager Web Software [36]. Due to the heterogeneity of data, quantitative synthesis was not possible.

### Measures of treatment effect

Data ascertained were heterogenous both in terms of study design and interventions undertake. As such, we were unable to estimate treatment effects. We described the included studies in the “Characteristics of included studies” table.

### Confidence in cumulative evidence

The quality of evidence was classified according to the Grading of Recommendations, Assessment, Development and Evaluation (GRADE) system into one of four categories: high, moderate, low, and very low [37]. Evidence based on randomized controlled trials was considered as high quality unless confidence in the evidence was decreased due to study limitations, the inconsistency of results, indirectness of evidence, imprecision, and reporting biases. Observational studies were considered low quality; however, they were graded higher if the treatment effect observed was very large or if there was evidence of a dose-response relationship [38, 39]. We have presented the evidence profile (EP) tables in the Appendix section.

## Results

### Results of the search

Our search resulted in 690 references without duplicates for screening (PRISMA diagram, Fig. 1). The title and abstract screening excluded further 499 studies. We screened the remainder of full-text studies. We attained further 18 full-text studies through gray literature searches. We included 10 full-text studies.

### Included studies

We included ten eligible studies. Please see the characteristics of included studies (Additional file 2). Five of these studies were simulation studies. Two of the studies were randomized controlled trials. A single study was a randomized controlled trial in a simulation setting [40]. A single study was an observational case series of healthcare workers (airway proceduralist only) managing patients infected with SARS-CoV-2 in China at the start of 2020 [41]. Two were observational studies with control group cohorts [42, 43]. One observational simulation study was a case series without a control group [44].

### Characteristics of participants

In the simulation studies, researchers included 195 participants. Two of the observational simulation studies were cross-over studies, and therefore, control participants were also intervention participants [42, 43, 45–48].

There were 153 participants in the randomized simulation studies. However, 24 of these acted as doffing observers in Andonian et al. study [49]. There were 1920 on-field healthcare workers performing intubations in two observational studies [41, 50].

### Interventions and comparisons

We identified a large prospective observational cohort study of healthcare workers utilizing a range of respiratory equipment including PAPR devices. The investigators reported that PAPRs (43.4%) were used more commonly in the United States of America (USA) than the United Kingdom (UK). In the UK participants more frequently used FFP3/N100 respirator masks (89.3%). The investigators did not report a significant difference in the primary endpoint rates in these two countries as determined by PPE use [50]. We identified a single retrospective observational case series which retrospectively assessed the rates of cross-infection in airway proceduralists. In both groups, HCWs utilized droplet precautions with either PAPR (*n* = 50); goggles, FFP2/N95 mask with a face shield (*n* = 22), or goggles, FFP/N95 with a full hood without positive pressure (*n* = 130) [41].

A single randomized controlled trial evaluated the effectiveness of training programs on the contamination of personal protective equipment incorporating PAPR [49]. A single observational study evaluated attitudes and practices towards a novel PAPR equipment [44]. A single observational study compared the effectiveness of different equipment including PAPR on donning and doffing [42]. A single observational study evaluated the effectiveness of different respiratory ensembles on the temperature of the skin and eye dryness [43]. A single simulation randomized prospective trial evaluated the PAPR versus E-RCP [40]. Three randomized simulation cross-over trials evaluated the impact of respiratory equipment including the use of PAPR on self-reported wearer comfort measures [45, 47, 48](Additional file 2).

### Outcomes

We identified a single prospective observational international multicenter cohort study (El-Boghdadly 2020) reporting the rates of assumed cross-infection with SARS-CoV-2 of healthcare personnel managing the airway. We identified a single observational case series (Yao 2020) which assessed the rates of cross-infection in anesthesiologists. In both groups, HCWs utilized droplet precautions with either PAPR (*n* = 50); goggles, FFP2/N95 mask with a face shield (*n* = 22), or goggles, FFP/N95 with a full hood without positive pressure (*n* = 130) [41]. We identified no studies assessing the efficacy of PAPR technology compared to alternative respirator/facepiece during care for patients with SARS-Cov-1, Ebola, or MERS.

We identified a single randomized cross-over trial (Zamora et al. 2006) which evaluated contamination of the skin or clothing measured with any type of test material to visualize contamination [40]; the identified study used fluorescein staining to measure contamination. Twenty-six percent of participants were contaminated in the PAPR group compared to 96% of contaminated participants in the E-RCP (enhanced respiratory controlled protection) group. We identified a single observational study [42]. In a single study (Chughtai et al. 2018) which evaluates the risk of contamination with different PPE and respiratory equipment, no participants using PAPR were contaminated. All participants using N95 were contaminated. We found no studies which assessed compliance with guidance on the selection of type and use of PPE measured with, e.g., observation checklist. We found three observational studies which evaluated the level of wearer comfort, and audibility with using the PAPR over alternative respiratory protection (Chughtai et al. 2018, Chughtai et al. 2020, Powell 2017) [42–44].

Three simulation cross-over randomized trials studied the use of PAPR versus APR, with the outcomes of wearer comfort as measured by user rating of mobility, ease of communication, ease of breathing, and heat perception [38, 45, 47, 48]. We identified no studies which evaluated the costs of the resource use including maintenance and cleaning of equipment. We identified a single randomized trial which evaluated the utility of training on donning and doffing of personal protective equipment including PAPR (Andonian et al. 2019) [49]. Structured training using a PAPR decreased the likelihood of self-contamination from 100 to 86%.

### Risk of bias

**Fig. 2:**
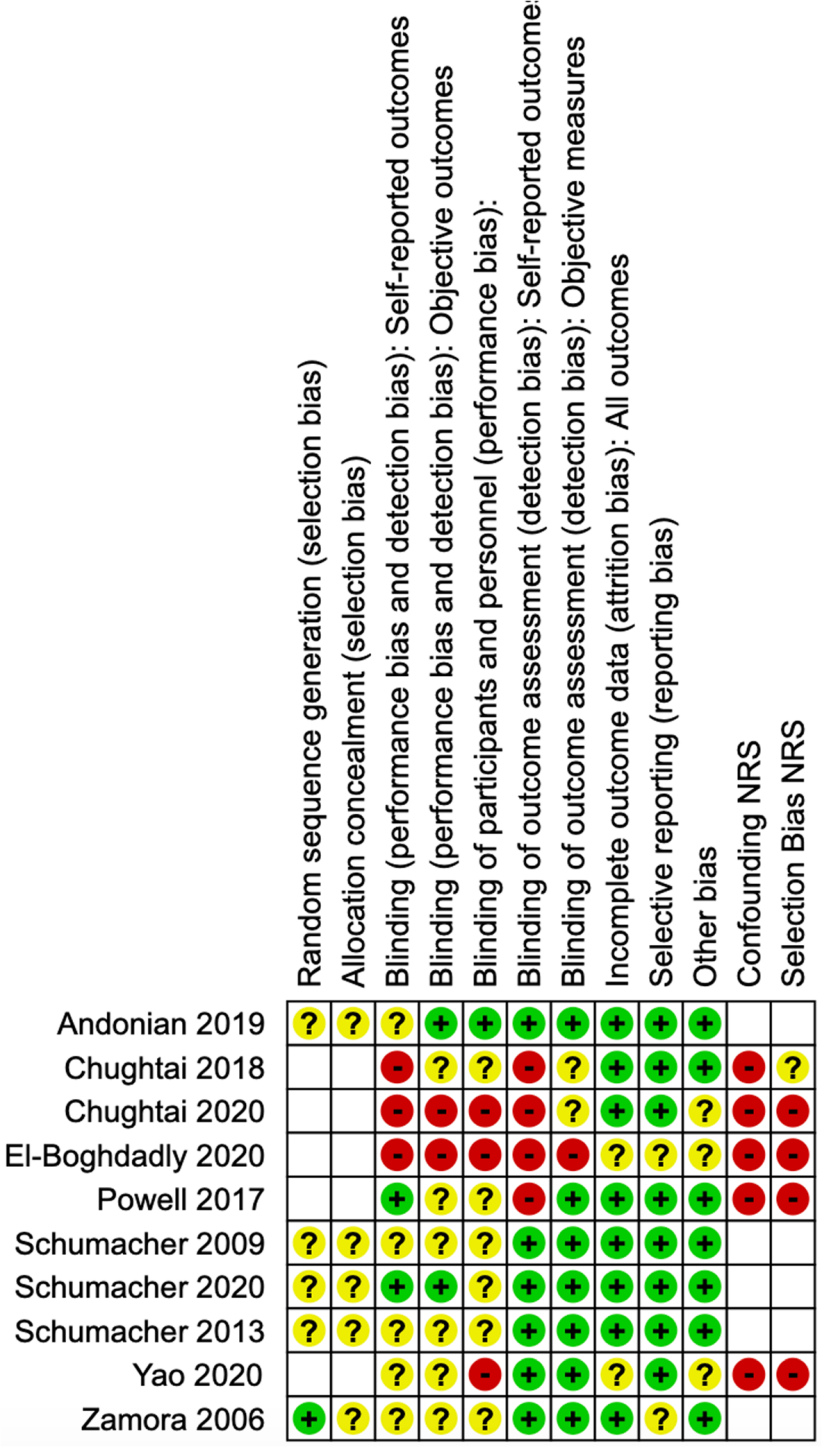
Risk of bias summary

**Fig. 3:**
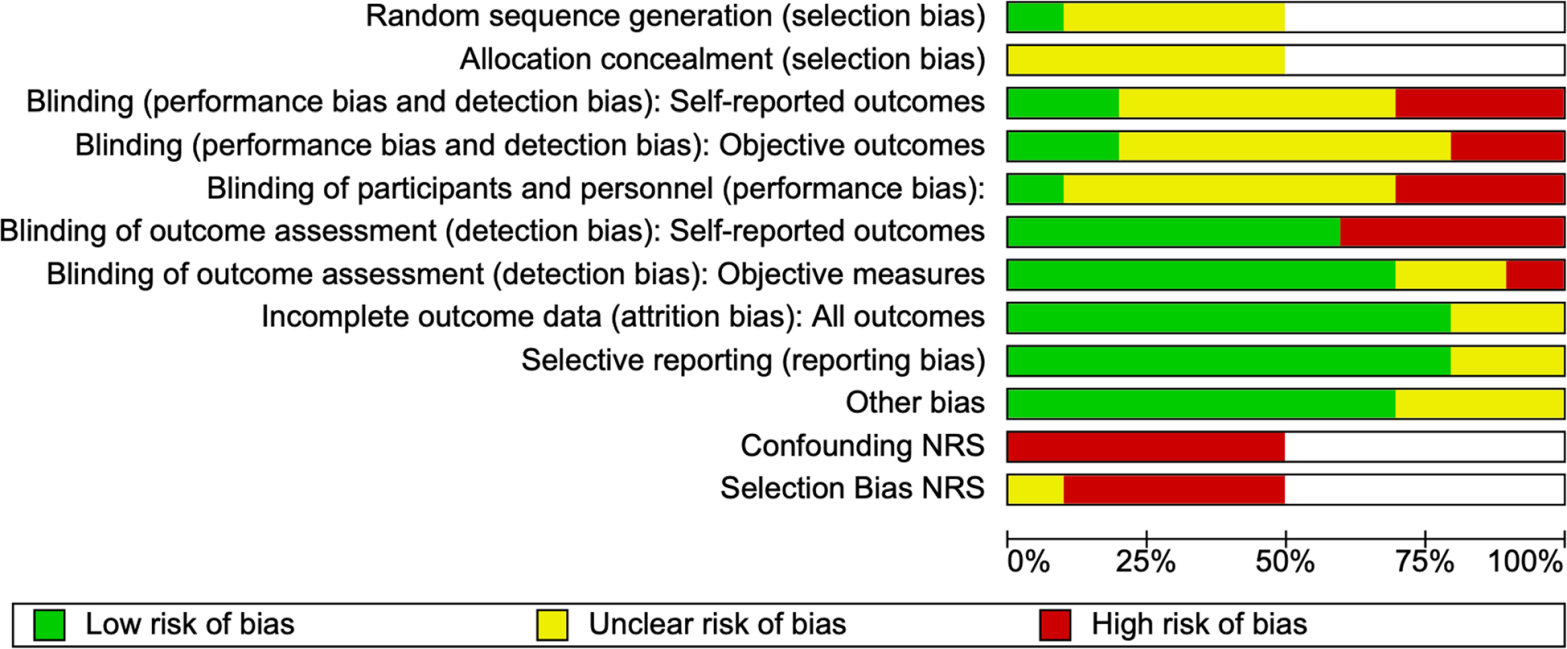
Risk of bias graph

We produced a risk of bias summary and a risk of bias graph for individual randomized and observational studies (Figs. 2 and 3). For non-randomized studies (NRS), we identified a high risk of bias across the confounding, selection bias, and blinding of outcome assessment across objective and subjective domains. For NRS studies, we identified an unclear risk of bias across the blinding performance bias and detection bias, objective outcomes. We used the risk of bias of individual studies to inform our assessment of bias across outcomes. Randomized controlled studies had an unclear risk of bias across a number of domains including allocation concealment, blinding of objective outcome assessment, and blinding of participants and personnel.

### Data synthesis

We summarized our findings in evidence profile (Additional file 3) tables across pre-determined primary and secondary outcomes using the GRADEpro software [51]. We performed a narrative synthesis of the data.

Data collected were not suitable for a meta-analysis due to inherent heterogeneity. There was no difference in the primary endpoint of COVID-19 infection in respective observational studies in the airway proceduralists utilizing PAPR versus other protective respiratory equipment [50]. In the prospective observational study, the primary endpoint was defined as the incidence of laboratory-confirmed COVID-19 diagnosis or new symptoms requiring self-isolation or hospitalization after a tracheal intubation episode. The overall incidence of the primary endpoint was 10.7% over a median follow-up of 32 days. Most participants were diagnosed through reported symptomatic self-isolation 144 (8.4%). The risk of the primary endpoint varied by country and was higher in females. The risk of COVID outcome was not associated with respiratory protection program use or use of PAPR [50]. Investigators did not report the exact number of users protected by PAPR devices. Consequently, we did not construct an EP table for this primary outcome. In the second observational study, there were no airway proceduralists who were cross-infected in either cohort. The rate of healthcare worker infection was significantly different in the two studies, 10.7% versus 0%. Contamination of the skin or clothing measured with any type of a test material yielded a lower risk of contamination in simulation studies. Evidence base for this outcome was low [40, 42].

There was moderate quality of evidence with regard to a lower risk of heat build-up in users with PAPR technology [45, 47]. There was a moderate quality of evidence that visibility was improved in PAPR in comparison with APR [45]. There was consistent moderate quality of evidence of decreased user rating of mobility and audibility with the use of PAPR [45, 47, 48]. In a single cohort observational study, all participants using N95 reported discomfort [42]. Powell et al. noted a lower temperature measurement in subjects using PAPR [43]. This did not translate to a self-reported greater level of comfort in this study.

Participants in a randomized study rated the ease of breathing with the PAPR system significantly better than with the APR [48].

## Discussion

Recently published field studies of HCWs managing patients with COVID-19 demonstrated equivalent rates of healthcare provider infection in cohorts utilizing PAPR versus other appropriate respiratory protection. We identified a trend towards a lower level of cross-contamination in participants using PAPR technology compared to alternative respiratory protection in low-quality simulation studies. We identified moderate quality of evidence towards improved healthcare worker comfort (heat tolerance and visibility) with PAPR technology compared with alternative respirators. PAPR users scored the technology lower with on mobility, dexterity, audibility, and communication. We identified moderate quality of evidence towards improved healthcare worker comfort (audibility and mobility) with APR (airflow-powered respirator) technology compared with PAPR.

There appears to be no reported difference in observed infection rates in participants utilizing PAPR or other appropriate respiratory protection. The preferred use of PAPR for respiratory protection may be due to perceived logistical advantages by institutional policy makers. A prospective international multicenter cohort study found no difference in infection rates between cohorts utilizing varied respiratory protection [50]. A series published recently found no airway proceduralist infections in the cohort utilizing PAPR versus a cohort equipped with more routine respiratory protection in addition to usual PPE [41]. This study was performed retrospectively in Wuhan during the outbreak of SARS-CoV-2 [41]. Differences in the airway proceduralist’s COVID outcomes appear distinct: 10.7% in the El-Boghdadly et al. study versus 0% reported in Yao et al. [41, 50]. These findings may have been confounded by a well-designed enhanced respiratory and contact protective system in the study with no provider infections.

We observed a trend towards lower contamination rates in simulation studies in participants utilizing PAPR [40, 42]. These observations are counterintuitive towards an assumption that due to the complexity of technology, cross-contamination during doffing with PAPR is more likely. The results of our review demonstrate a trend towards lower HCW contamination rates and decreased doffing violations whilst utilizing PAPR.

We found no studies which assessed compliance with donning or doffing protocols for equipment utilizing PAPR.

In line with subjective reports that PAPR may be more effective in decreasing the effort needed to maintain the work of breathing compared to a more conventional filtering facepiece, we identified moderate quality of evidence for this outcome [7, 43]. We identified a moderate quality of evidence towards improved healthcare worker comfort with regard to heat tolerance and visibility with PAPR technology. It is thought that through the positive airflow, PAPR’s eliminated the heat build-up [52]. A decrease in audibility and communication difficulties can be anticipated due to increased weight of the equipment and noise generated by positive airflow. In observational studies, we identified a trend towards a greater level of self-reported comfort amongst the PAPR wearers [42, 44]. Powell et al. noted a lower temperature measurement in subjects using PAPR [43]. Prior reports have outlined the potential for claustrophobia in healthcare providers with field use of PAPR [53]. During the tuberculosis outbreaks, the use of PAPR’s had a low institutional uptake. This occurred due to a number of factors, including concerns that doctors would appear frightening to their patients and that the motor’s hissing noise would interfere with patient communication [54]. The greater acceptance of PAPR by HCWS during both the SARS-Cov-1 pandemic and Ebola may be influenced by HCW perception of relative risk. Khoo et al. published a survey illustrating that PAPR as opposed to N95 was more comfortable for HCWs during an Ebola outbreak in Singapore [55].

We identified no studies exploring the costs of the resource use of PAPR versus any other filtration pieces. The costs of maintenance of PAPR equipment which require disinfection, cleaning, self-storage, battery maintenance, and a requirement for education of a significant proportion of the HCW workforce have not been considered in evidence-based literature. These costs are juxtaposed against more wears per piece of PAPR compared to disposable face-filtering pieces. The PAPR use may be a resource utilization prepared strategy for times of a greater need for N95/FFP2. It has been noted that there have been fewer equipment shortages for PAPR than N95 [56].

We identified a single simulation randomized controlled trial which demonstrated a trend towards a lower risk of contamination when the PAPR use was incorporated with a teaching program. During the SARS-CoV-1 outbreak, recent training in infection control increased the likelihood of workers’ adherence to recommended barrier precautions [57]. Whilst the initial focus was on the use of more stringent respiratory PPE components, further studies found that SARS-CoV-1 transmission was not supported if more standardized PPE was used. Critical system factors protecting the HCWs included compliance with N95 mask application and ongoing use, as well as complementary respiratory protection protocols [25].

Current reports of the choice of protective respiratory technology during the SARS-CoV-2 pandemic are disparate. In a recently published experience of intubation and ventilation of critically ill patients in Wuhan, Meng et al. illustrated the use of a positive pressure ventilation system for anesthesiologists dealing with COVID-19-positive patients [58]. There have been three separate descriptive reports from Singapore on the routine use of PAPR in their protocols for anesthesia in suspected or confirmed COVID-19 patients [59, 60] [27]. Recommendations from the Joint Task Force of the Chinese Society of Anesthesiology and the Chinese Association of Anesthesiologists center on the N95 use for proceduralists. These recommendations do not specifically mention the use of a PAPR device. Although some authors make recommendations for the use of PAPR for critical care of COVID-19 patients, they acknowledge that there is no conclusive evidence to show that this advanced respiratory technology decreases the likelihood of viral airborne transmission [61].

The utilization of PAPR with high filtration efficiency may represent an example of a “precautionary principle” wherein the action taken to reduce the risk is guided by logistical advantages of the PAPR system. With a higher APF factor than N95 masks, it is scientifically plausible that the PAPR use may result in a long-term lower HCW infection rates. There is however limited literature supporting the PAPR use during epidemics/pandemics of SARS-CoV-1, SARS-CoV-2, MERS, and Ebola. Given the lack of demonstrable efficacy, institutional decision makers may be applying a pragmatic choice to use PAPR on the basis of the precautionary principle.

Current PAPR certification standards have been developed primarily for industrial applications. There is a need for respirator standards to better expand to suit the requirements of healthcare workers [62]. In terms of the laboratory research, industrial, radioactive, or biological particles behave in a similar manner with regard to a filtration standard. The quantification of the infectious dose with this emerging viral disease has not occurred. Therefore, it remains a challenge to determine the optimum respiratory protection under individual circumstances. Future developments include adjusting the testing standards to activities to which the user (HCW) is engaged.

Our systematic review has been limited by a number of available studies graded as low evidence. A recently published study by El-Boghdadly et al. had only 28.8% of laboratory-confirmed infections. The remainder were diagnosed through self-isolation and hospitalization without confirmed laboratory testing [50]. In addition, in the absence of phylogenetic analysis, it is not possible to conclude the source of infection, be it patient contact or community-acquired. The comparison of infection rates with HCWs not wearing the PAPR technology may be biased by other PPE protection factors such as the utility of system-related compliance measures [63]. Despite the theoretical advantages of PAPR, there have to date been no controlled clinical trials on the efficacy of this technology during the SARS-Cov-1, SARS-Cov-2, EBOLA, or MERS pandemics in comparison with other high-level respiratory protection [64]. At present, the minimal infective dose for SARS-CoV-2 pathogen is unknown for any of the transmission modes [65]. Higher viral load shedding may be more readily associated with greater disease severity [66]. Whether a higher PAPR filtration factor translates to decreased infection rates of HCWs remains to be elucidated. True randomized controlled studies may not be ethically feasible due to the higher filtration factor of PAPR. Pragmatic observational studies, as published recently in well-resourced areas may be both more ethical and feasible [41]. Most of the studies included have been performed using simulation. Despite the simulation being designed to simulate exposure to highly contagious diseases, they are performed in a safe setting without true haste [46]. This may introduce systematic bias to the studies themselves and the review. We graded the risk of bias in observational on-field studies as high. This is due to a number of factors including the observational nature of SARS-CoV-2 infection rate assessment and potential for confounding due to attendant infection control processes.

## Conclusion

Equivalent rates of healthcare provider infection have been demonstrated in cohorts utilizing PAPR versus other appropriate respiratory protection. There have been no field studies reporting the effectiveness and utility of PAPR in protecting the healthcare workers from cross-infection due to other highly virulent viral diseases including SARS-CoV-1, Ebola, or MERS. Evidence base of low quality indicates greater wearer protection in HCWs using PAPR compared to alternative respiratory devices, from cross-contamination and during doffing in simulation studies. Provider satisfaction appears higher with regard to thermal comfort; however, lower in relation to audibility and mobility with PAPR technology. Precautionary principles may be guiding the institutional risk management strategies of HCW protection during epidemics and pandemics.

The closure of this knowledge gap with regard to optimal respiratory protection during pre-defined highly virulent pandemics needs further prospectively collected field data.

## Supplementary information

**Additional file 1.** Search strategy.

**Additional file 2.** Characteristics of included studies.

**Additional file 3.** Evidence profile tables.

## Data Availability

Not applicable

## Abbreviations

HCW: Healthcare worker
MERS-CoV: Middle East respiratory syndrome coronavirus
SARS: Severe acute respiratory syndrome
PPE: Personal protective equipment
WHO: World Health Organization
PAPR: Powered air-purifying respirator
