# Supplementary material for: Use of powered air-purifying respirator (PAPR) by healthcare workers for preventing highly infectious viral diseases—a systematic review of evidence
Source: Syst Rev. 2020 Aug 8;9:173. doi: 10.1186/s13643-020-01431-5 (PMC7414632; doi:10.1186/s13643-020-01431-5)
Supplement: Supplementary file 1 — Additional file 1. Search strategy. [file 13643_2020_1431_MOESM1_ESM.docx]

Database: Ovid MEDLINE(R) ALL <1946 to May 12, 2020>

Search Strategy:

--------------------------------------------------------------------------------

1 Respiratory Insufficiency/ or Pneumonia, Viral/ or Lung Diseases/ or Respiratory Protective Devices/ or Pressure Ulcer/ or Continuous Positive Airway Pressure/ or Humans/ or respiratory devices.mp. or Coronavirus Infections/

2 Masks/ or masks.mp.

3 1 or 2

4 E-RCP.mp.

5 exp Respiratory Protective Devices/ or filtering face piece.mp. or exp Masks/

6 N-95.mp.

7 FFP2.mp.

8 FFP3.mp.

9 air-purifying respirator.mp. or Respiratory Protective Devices/

10 powered air-purifying respirator.mp.

11 3 or 4 or 5 or 6 or 7 or 8 or 9 or 10

12 exp Health Personnel/

13 nurse.tw.

14 dentist.tw.

15 medical worker.tw.

16 ambulance.tw.

17 physio.tw.

18 physician.tw.

19 transport.tw.

20 12 or 13 or 14 or 15 or 16 or 17 or 18 or 19

21 11 and 21

22 Ebolavirus/ or Hemorrhagic Fevers, Viral/ or Hemorrhagic Fever, Ebola/ or ebola.mp.

23 Disease Transmission, Infectious/ or disease transmission.mp. or Communicable Diseases/

24 SARS Virus/ or Betacoronavirus/ or Severe Acute Respiratory Syndrome/

25 Coronavirus Infections/ or Severe Acute Respiratory Syndrome/ or SARS Virus/ or Pneumonia, Viral/ or Betacoronavirus/

26 SARS Virus/ or Pneumonia, Viral/ or Coronavirus Infections/ or SARS-CoV-2.mp. or Betacoronavirus/

27 MERS.mp. or Middle East Respiratory Syndrome Coronavirus/ or Coronavirus/

28 Coronavirus Infections/ or Pneumonia, Viral/ or human-to- human transmission.mp.

29 22 or 23 or 24 or 25 or 26 or 27 or 28

30 20 and 29
