# Supplementary material for: Use of powered air-purifying respirator (PAPR) by healthcare workers for preventing highly infectious viral diseases—a systematic review of evidence
Source: Syst Rev. 2020 Aug 8;9:173. doi: 10.1186/s13643-020-01431-5 (PMC7414632; doi:10.1186/s13643-020-01431-5)
Supplement: Supplementary file 2 — Additional file 2. Characteristics of included studies. [file 13643_2020_1431_MOESM2_ESM.docx]

*Characteristics of Included Studies*

| *Study* | *Virus studied or*  *Simulation* | *Study Design* | *Number of*  *Participants* | *Primary Outcomes*  *-PAPR focused* | *Secondary Outcomes*  *-PAPR*  *focused* | *Results* | *Level of Evidence* |
| --- | --- | --- | --- | --- | --- | --- | --- |
| Andonian at al 2019  (1) | Simulation of training on PPE doffing including PAPR | Randomised Controlled Trial | 48  (13 HCW in intervention group and 13 HCW control group)  -others randomised to Doffing Assistants | 1.Effectiveness of training on contamination of a number of body sites | 2. Effectiveness of training on contamination using ultraviolet light and PLS swabbing | -11/13 HCW contaminated at least one body site;  -13/13 HCW contaminated at least one body site;  -median contamination score lower in the intervention group: 23.15 vs 64.45 p=0.004 (ultraviolet light);  - median contamination score lower in the intervention group:72.4 vs144.8 p=0.001 (PLS visualization); | 2 |
| Chughtai et al  2020  (2) | Simulation by fit-testing and use of PAPR | Observational | 20 | 1.Examine attitudes and practices regarding PAPR | 2. Determine acceptability of a novel PAPR (CleanSpace2^tm^) | -14/20 participants found PAPR easy to don;  -15/20 participants found PAPR easy to doff;  -14/20 participants found PAPR comfortable to wear; | 4 |
| Chughtai et al 2018  (3) | Simulation of doffing protocols including PAPR | Observational | 30 PPE sequences tested, 6 including PAPR  (10 participants) | 1.Evaluate risk of self- contamination using ultraviolet light | 2.Evaluate user level of comfort  Including breathability and ease of breathing | -4/24 participants contaminated using N95;  -0/6 participants contaminated using PAPR;  -24/24 reported discomfort with N/95 use;  -2/6 reported discomfort with PAPR use; | 3 |
| El-Boghdadly et al 2020  (4) | Field study  of SARS-CoV-2 | Observational  -prospective international multicentre cohort study | -1718 healthcare workers reported 5148 tracheal  intubation episodes;  -exact protective equipment composition not reported but included N95/P2/P3/  PAPR | The primary endpoint was the incidence of laboratory-confirmed COVID-19 diagnosis or  new symptoms requiring self-isolation or hospitalization after a tracheal intubation episode. | N/A | -overall incidence of COVID outcomes was 10.7% over a median follow up of 32 days;  -risk of the primary endpoint varied by country and gender, higher in females; | 3 |
| Powell et al  2017  (5) | Simulation assessment of participant comfort | Observational | 60 total assessments  (12 participants)  -12 N95;  -12 tight fit PAPR;  -24 loose PAPR;  -12 hybrid PAPR;  (note hybrid PAPR excluded from analysis) | 1. Evaluate face and body thermal sensations; | 2. Evaluate wearer comfort through assessment of eye dryness; | -temperature of facial skin lower in 36 PAPR compared to N95;  -tight fitting face piece PAPR increased eye dryness;  -loose fitting PAPR did not increase eye dryness;  -perception of comfort equivalent in two groups;  -perception of work of breathing was equivalent in the two groups; | 3 |
| Schumacher  et al 2009  (6) | Simulation study of impact of respiratory  protective  equipment during emergency life support | Randomised cross-over  simulation study | 42 total assessments  (14 paramedics)  -three types of respiratory equipment:  1.PAPR  (positive pressure)  2. Standard APR ( negative pressure)  3. Standard surgical protection  (control group) | 1.Difference in treatment times in two resuscitation scenarios, with standard equipment use serving as control | 2. Wearer comfort as measured by user rating of  -mobility  -ease of communication/audibility  -ease of breathing | -The participating paramedics rated the ease of breathing with the PAPR system significantly better than with the APR.  (p<0/05);  -The wearer comfort in respect of mobility and the ability to communicate was similar in both respirator groups; | 2 |
| Schumacher  et al 2013  (7) | Simulation study of impact of respiratory  protective  equipment during emergency paediatric support | Randomised cross-over  simulation study | 48 total assessments  (16 paramedics)  -three types of respiratory equipment:  1.PAPR  (positive pressure)  2. Standard APR ( negative pressure)  3. Standard surgical protection  (control group) | 1.Difference in treatment times in two resuscitation scenarios, with standard equipment use serving as control | 2.Wearer comfort as measured by user rating of  -mobility  -noise/speech intelligibility  -heat | -Study subjects reported that communication (p=0.001) and mobility (p=0.000) were significantly improved in the APR group compared to PAPR;  -Study subjects reported that heat-build-up was significantly less in the PAPR-hood group (p=000); | 2 |
| Schumacher  2020  (8) | Simulation study of impact of respiratory  protective  equipment on airway  management | Randomised cross-over  simulation study | 300 total assessments  (25 anaesthetists)  -three types of respiratory equipment:  1.PAPR  (positive pressure)  2. Standard APR ( negative pressure)  3. Standard surgical protection  (control group)  -Four different intubation drills | 1.Difference in  Intubation times in different airway scenarios | 2.Wearer comfort as measured by user rating of  -mobility  -noise  -heat  -vision  -speech intelligibility | The powered respirator ensemble scored significantly better in user rating for  -heat (p=0.002)  -vision (p=0.008)  The powered respirator ensemble scored significantly worse in user rating for  -noise (p=0.021)  -speech intelligibility (p=0.062) | 2 |
| Yao et al  2020  (9) | Field study  of SARS-CoV-2 | Retrospective observational case series of two centres | 1.outer layer with full PAPR n=50;  2.1.outer layer with goggles, FFP2/N95 with a face shield n=22;  2.2 outer layer with goggles, FFP/N95 with a full hood without positive pressure n=130; | Analysis of two centre data by a panel of international experts where  both groups wore an inner layer of protection in addition to designated protective equipment;  A number of recommendations were made on the analysis of data and opinion on experts; | | -zero transmission rates were noted in both groups;  -larger series is needed to give greater confidence;  -experts noted that outcome data, or the association between level of PPE and coronavirus transmission from the current epidemic are lacking; | 3 |
| Zamora et al  2006  (10) | Simulation assessment of contamination | Prospective randomised controlled cross over study | 50 participants  (PAPR versus E-RCP) | 1.Evaluate any contamination, size of contamination area; | 2.Donning non-compliance;  3.Doffing non-compliance; | -13/50 any contamination PAPR group;  -48/50 any contamination E-RCP group;  -15/50 donning non-compliance PAPR;  -2/50 donning non-compliance E-RCP;  -6/50 doffing non-compliance PAPR  -12/50 doffing non-compliance E-RCP; | 2 |

Studies:

1. Andonian J, Kazi S, Therkorn J, Benishek L, Billman C, Schiffhauer M, et al. Effect of an Intervention Package and Teamwork Training to Prevent Healthcare Personnel Self-contamination During Personal Protective Equipment Doffing. Clinical infectious diseases : an official publication of the Infectious Diseases Society of America. 2019;69(Supplement_3):S248-S55.

2. Chughtai AA, Seale H, Rawlinson WD, Kunasekaran M, Macintyre CR. Selection and Use of Respiratory Protection by Healthcare Workers to Protect from Infectious Diseases in Hospital Settings. Ann Work Expo Health. 2020;64(4):368-77.

3. Chughtai AA, Chen X, Macintyre CR. Risk of self-contamination during doffing of personal protective equipment. American journal of infection control. 2018;(no pagination).

4. El-Boghdadly K, Wong DJN, Owen R, Neuman MD, Pocock S, Carlisle JB, et al. Risks to healthcare workers following tracheal intubation of patients with COVID-19: a prospective international multicentre cohort study. Anaesthesia. 2020.

5. Powell JB, Kim JH, Roberge RJ. Powered air-purifying respirator use in healthcare: Effects on thermal sensations and comfort. J Occup Environ Hyg. 2017;14(12):947-54.

6. Schumacher J, Gray SA, Weidelt L, Brinker A, Prior K, Stratling WM. Comparison of powered and conventional air-purifying respirators during simulated resuscitation of casualties contaminated with hazardous substances. Emergency medicine journal. 2009;26(7):501‐5.

7. Schumacher J, Gray SA, Michel S, Alcock R, Brinker A. Respiratory protection during simulated emergency pediatric life support: a randomized, controlled, crossover study. Prehospital and disaster medicine. 2013;28(1):33‐8.

8. Schumacher J, Arlidge J, Dudley D, Sicinski M, Ahmad I. The impact of respiratory protective equipment on difficult airway management: a randomised, crossover, simulation study. Anaesthesia. 2020.

9. Yao W, Wang T, Jiang B, Gao F, Wang L, Zheng H, et al. Emergency tracheal intubation in 202 patients with COVID-19 in Wuhan, China: lessons learnt and international expert recommendations. British Journal of Anaesthesia.

10. Zamora JE, Murdoch J, Simchison B, Day AG. Contamination: a comparison of 2 personal protective systems. CMAJ. 2006;175(3):249-54.
