# Supplementary material for: Use of powered air-purifying respirator (PAPR) by healthcare workers for preventing highly infectious viral diseases—a systematic review of evidence
Source: Syst Rev. 2020 Aug 8;9:173. doi: 10.1186/s13643-020-01431-5 (PMC7414632; doi:10.1186/s13643-020-01431-5)
Supplement: Supplementary file 3 — Additional file 3. Evidence profile tables. [file 13643_2020_1431_MOESM3_ESM.docx]

**Primary Outcome: 2.** Contamination of skin or clothing measured with any type of test material to visualize contamination;

**Question**: PAPR compared to E-RCP for respiratory protection in healthcare workers dealing with patients infected with highly virulent viral diseases?

**Setting**: Aerosol Generating Procedures or Prolonged Contact with Infected Patients

**Bibliography**: Zamora 2006(1) Chughtai 2018 (2);

| **Certainty assessment** | | | | | | | **№ of patients** | | **Effect** | | **Certainty** | **Importance** |
| --- | --- | --- | --- | --- | --- | --- | --- | --- | --- | --- | --- | --- |
| **№ of studies** | **Study design** | **Risk of bias** | **Inconsistency** | **Indirectness** | **Imprecision** | **Other considerations** | **PAPR** | **E-RCP** | **Relative (95% CI)** | **Absolute (95% CI)** |  |  |
| **Any contamination** | | | | | | | | | | | | |
| 1  Zamora et al 2006 | randomised trials  (Zamora2006) | serious | serious | serious | serious | all plausible residual confounding would suggest spurious effect, while no effect was observed | 13/50 (26.0%) | 48/50 (96.0%) | **RR 0.27** (0.17 to 0.43) | **701 fewer per 1,000** (from 797 fewer to 547 fewer) | ⨁◯◯◯ VERY LOW | IMPORTANT |
| **Contamination greater than 1 cm** | | | | | | | | | | | | |
| 1 | randomised trials  (Zamora2006) | serious | serious | serious | serious | all plausible residual confounding would suggest spurious effect, while no effect was observed | 10/50 (20.0%) | 48/50 (96.0%) | **RR 0.21** (0.12 to 0.36) | **758 fewer per 1,000** (from 845 fewer to 614 fewer) | ⨁◯◯◯ VERY LOW | IMPORTANT |
| **Size of the contamination area** | | | | | | | | | | | | |
| 1 | randomised trials  (Zamora2006) | serious | serious | serious | serious | all plausible residual confounding would suggest spurious effect, while no effect was observed | 50 | 50 | - | mean **81.1 lower** (96.07 lower to 66.13 lower) | ⨁◯◯◯ VERY LOW | IMPORTANT |
| **Donning non-compliance** | | | | | | | | | | | | |
| 1 | randomised trials  (Zamora2006) | serious | serious | serious | serious | all plausible residual confounding would suggest spurious effect, while no effect was observed | 15/50 (30.0%) | 2/50 (4.0%) | **RR 7.50** (1.81 to 31.10) | **260 more per 1,000** (from 32 more to 1,000 more) | ⨁◯◯◯ VERY LOW | IMPORTANT |
| **Doffing non-compliance** | | | | | | | | | | | | |
| 1 | randomised trials  (Zamora2006) | serious | serious | serious | serious | all plausible residual confounding would suggest spurious effect, while no effect was observed | 6/50 (12.0%) | 12/50 (24.0%) | **RR 0.50** (0.20 to 1.23) | **120 fewer per 1,000** (from 192 fewer to 55 more) | ⨁◯◯◯ VERY LOW | IMPORTANT |
| **Any contamination** | | | | | | | | | | | | |
| 1 | observational studies  (Chughtai2018) | serious | serious | serious | serious | all plausible residual confounding would suggest spurious effect, while no effect was observed | 0/6 (0.0%) | 4/24 (16.7%) | not estimable |  | ⨁◯◯◯ VERY LOW |  |

**CI:** Confidence interval; **RR:** Risk ratio

**Secondary Outcomes: 1.** level of wearer comfort, visibility and audibility whilst using the PAPR over alternative respiratory protection;

**2.** objective and/or subjective measures of work of breathing during the use of PAPR versus alternative respiratory protective equipment;

**Question**: PAPR compared to other respiratory protection for infection control methods for level of wearer comfort

**Question: What is the level of wearer comfort with PAPR**

**Setting**: Aerosol Generating Procedures or Prolonged Contact with Infected Patients

**Bibliography**: Chughtai et al 2020 (3), Chughtai et al 2018 (2), Powell et al 2017, (4) Scumacher et al 2020, Scumacher et al 2013 , Schumacher et al 2009 (5-8)

| **Certainty assessment** | | | | | | | **№ of patients** | | **Effect** | | **Certainty** | **Importance** |
| --- | --- | --- | --- | --- | --- | --- | --- | --- | --- | --- | --- | --- |
| **№ of studies** | **Study design** | **Risk of bias** | **Inconsistency** | **Indirectness** | **Imprecision** | **Other considerations** | **PAPR** | **other respiratory protection** | **Relative (95% CI)** | **Absolute (95% CI)** |  |  |
| **Comfort of donning PAPR** | | | | | | | | | | | | |
| 1  Chughtai et al 2020 | observational studies | extremely serious | serious | serious | serious | all plausible residual confounding would reduce the demonstrated effect | 14/20 (70.0%) | - | - | - | ⨁◯◯◯ VERY LOW | IMPORTANT |
| **Comfort of doffing PAPR** | | | | | | | | | | | | |
| 1  Chughtai et al 2018 | observational studies | extremely serious | serious | serious | serious | strong association all plausible residual confounding would suggest spurious effect, while no effect was observed | 15/20 (75.0%) | 0.0% | not estimable |  | ⨁◯◯◯ VERY LOW | IMPORTANT |
| **Level of reported wearer discomfort** | | | | | | | | | | | | |
| 2  Chughtai et al 2020  Chughtai et al 2018 | observational studies | extremely serious | serious | serious | serious | all plausible residual confounding would suggest spurious effect, while no effect was observed | 8/30 (26.7%) | 24/24 (100.0%) | not estimable |  | ⨁◯◯◯ VERY LOW | IMPORTANT |
|  |  |  |  |  |  |  |  | 0.0% |  |  |  |  |
| **Level of wearer comfort on a self reported scale** | | | | | | | | | | | | |
| 1  Powell et 2017 | observational studies | serious | serious | serious | serious | all plausible residual confounding would suggest spurious effect, while no effect was observed | 36 | 12 | - | **0**  (0 to 0 ) | ⨁◯◯◯ VERY LOW |  |
| **Self reported work of breathing** | | | | | | | | | | | | |
| 1  Powell et al 2017 | observational studies | serious | serious | serious | serious | all plausible residual confounding would suggest spurious effect, while no effect was observed | 36 | 12 | - | **0**  (0 to 0 ) | ⨁◯◯◯ VERY LOW |  |
| **Facial temperature as a surrogate measure of wearer comfort** | | | | | | | | | | | | |
| 1  Powell et al 2017 | observational studies | serious | serious | serious | serious | all plausible residual confounding would suggest spurious effect, while no effect was observed | 36 | 12 | - | **0**  (0 to 0 ) | ⨁◯◯◯ VERY LOW |  |
| **Self-reported perception of heat build up as a measure of wearer comfort** | | | | | | | | | | | | |
| 2  Schumacher et al 2013  And Schumacher 2020 | randomised trials | serious | not serious | serious | not serious | all plausible residual confounding would suggest spurious effect, while no effect was observed | 116 | 232 | - | **0**  (0 to 0 ) | ⨁⨁⨁◯ MODERATE |  |
| **Self-reported perception of visibility as a measure of wearer comfort** | | | | | | | | | | | | |
| 1  Schumacher et al 2020 | randomised trials | serious | not serious | serious | not serious | all plausible residual confounding would reduce the demonstrated effect | 100 | 200 | - | **0**  (0 to 0 ) | ⨁⨁⨁◯ MODERATE |  |
| **Self-reported perception of audibility/communication** | | | | | | | | | | | | |
| 3  Schumacher al 2020  Schumacher et al 2013  Schumacher et al 2009 | randomised trials | serious | not serious | serious | not serious | all plausible residual confounding would reduce the demonstrated effect | 130 | 260 | - | **0**  (0 to 0 ) | ⨁⨁⨁◯ MODERATE |  |
| **Self-reported perception of mobility as a measure of wearer comfort** | | | | | | | | | | | | |
| 2  Schumacher et al 2013  Schumacher et al 2009 | randomised trials | serious | not serious | serious | not serious | all plausible residual confounding would suggest spurious effect, while no effect was observed | 30 | 60 | - | **0**  (0 to 0 ) | ⨁⨁⨁◯ MODERATE |  |
| **Self reported work of breathing** | | | | | | | | | | | | |
| 1  Schumacher et al 2009 | randomised trials | serious | not serious | serious | not serious | all plausible residual confounding would suggest spurious effect, while no effect was observed | 14 | 28 | - | **0**  (0 to 0 ) | ⨁⨁⨁◯ MODERATE |  |

**CI:** Confidence interval

**Secondary Outcome: 4.** impact of structured training programs on PAPR use over alternative training or no teaching;

**Question**: Should structured training in PPE( including PAPR) compared to no structured training be implemented for Healthcare workers?

**Setting**: Perioperative patient care

**Bibliography**: Andonian 2019 (9)

| **Certainty assessment** | | | | | | | **№ of patients** | | **Effect** | | **Certainty** | **Importance** |
| --- | --- | --- | --- | --- | --- | --- | --- | --- | --- | --- | --- | --- |
| **№ of studies** | **Study design** | **Risk of bias** | **Inconsistency** | **Indirectness** | **Imprecision** | **Other considerations** | **structured training in PPE( including PAPR)** | **no structured training** | **Relative (95% CI)** | **Absolute (95% CI)** |  |  |
| **Number of Healthworkers contaminating at least one body surface with fluorescein** | | | | | | | | | | | | |
| 1  Andonian et al 2019 | randomised trials | serious | serious | serious | serious | all plausible residual confounding would suggest spurious effect, while no effect was observed | 11/13 (84.6%) | 13/13 (100.0%) | not estimable |  | ⨁◯◯◯ VERY LOW | IMPORTANT |

**CI:** Confidence interval; **RR:** Risk ratio

1. Zamora JE, Murdoch J, Simchison B, Day AG. Contamination: a comparison of 2 personal protective systems. CMAJ. 2006;175(3):249-54.

2. Chughtai AA, Chen X, Macintyre CR. Risk of self-contamination during doffing of personal protective equipment. American journal of infection control. 2018;(no pagination).

3. Chughtai AA, Seale H, Rawlinson WD, Kunasekaran M, Macintyre CR. Selection and Use of Respiratory Protection by Healthcare Workers to Protect from Infectious Diseases in Hospital Settings. Ann Work Expo Health. 2020;64(4):368-77.

4. Powell JB, Kim JH, Roberge RJ. Powered air-purifying respirator use in healthcare: Effects on thermal sensations and comfort. J Occup Environ Hyg. 2017;14(12):947-54.

5. Laboratory performance evaluation of N95 filtering facepiece respirators, 1996. MMWR Morb Mortal Wkly Rep. 1998;47(48):1045-9.

6. Schumacher J, Arlidge J, Dudley D, Sicinski M, Ahmad I. The impact of respiratory protective equipment on difficult airway management: a randomised, crossover, simulation study. Anaesthesia. 2020.

7. Schumacher J, Gray SA, Michel S, Alcock R, Brinker A. Respiratory protection during simulated emergency pediatric life support: a randomized, controlled, crossover study. Prehospital and disaster medicine. 2013;28(1):33‐8.

8. Schumacher J, Gray SA, Weidelt L, Brinker A, Prior K, Stratling WM. Comparison of powered and conventional air-purifying respirators during simulated resuscitation of casualties contaminated with hazardous substances. Emergency medicine journal. 2009;26(7):501‐5.

9. Andonian J, Kazi S, Therkorn J, Benishek L, Billman C, Schiffhauer M, et al. Effect of an Intervention Package and Teamwork Training to Prevent Healthcare Personnel Self-contamination During Personal Protective Equipment Doffing. Clinical infectious diseases : an official publication of the Infectious Diseases Society of America. 2019;69(Supplement_3):S248-S55.
